# Suppression of type I interferon production by human T-cell leukemia virus type 1 oncoprotein Tax

**DOI:** 10.1186/1742-4690-11-S1-P77

**Published:** 2014-01-07

**Authors:** Dong-Yan Jin, Kin-Hang Kok, Hei-Man V Tang, Pak-Yin Lui, Chi-Ping Chan

**Affiliations:** 1 Department of Biochemistry, The University of Hong Kong, Pokfulam, Hong Kong

## 

Human T-cell leukemia virus type 1 (HTLV-1) is the etiologic agent of adult T-cell leukemia (ATL) and tropical spastic paraparesis. Type I interferons are key effectors of innate antiviral response and have been tested as anti-HTLV-1 and anti-ATL agents. HTLV-1 oncoprotein Tax is known to suppress innate interferon response by activating SOCS1, an inhibitor of interferon signaling that activates the expression of interferon-stimulated genes. Whether Tax might also suppress interferon production remains to be determined. Here we report on the suppression of type I interferon production by HTLV-1 Tax through interaction with and inhibition of TBK1 kinase. HTLV-1-transformed ATL cells were found to be unable to boost the production of type I interferons in response to Sendai virus infection. This inability was attributed to Tax. Expression of Tax alone sufficiently repressed the induction of interferon production by RIG-I + PACT, TBK1 and IRF3, but not by IRF3-5D, a dominant active phosphomimetic mutant. This suggests that Tax might act at a point prior to IRF3 phosphorylation. Reciprocal co-immunoprecipitation and immunoblotting experiments confirmed the association of Tax with TBK1 kinase that phosphorylates IRF3. In vitro kinase assay indicated an inhibitory effect of Tax on TBK1-mediated phosphorylation of IRF3. Taken together, our findings suggested a new mechanism by HTLV-1 oncoprotein Tax circumvents the production of type I interferons in infected cells. (Supported by HKU7661/08M, HKU7674/12M, HKU1/CRF/11G and SKY-MRF-2011).

